# Prediction of Protein-DNA Interface Hot Spots Based on Empirical Mode Decomposition and Machine Learning

**DOI:** 10.3390/genes15060676

**Published:** 2024-05-23

**Authors:** Zirui Fang, Zixuan Li, Ming Li, Zhenyu Yue, Ke Li

**Affiliations:** 1 School of Information and Artificial Intelligence, Anhui Agricultural University, Hefei 230036, China; 21115126@stu.ahau.edu.cn (Z.F.); lizx@stu.ahau.edu.cn (Z.L.); 21115141@stu.ahau.edu.cn (M.L.);; 2 Anhui Provincial Engineering Laboratory for Beidou Precision Agriculture Information, Anhui Agricultural University, Hefei 230036, China; 3 Institute of Artificial Intelligence, Hefei Comprehensive National Science Center, Hefei 230088, China

**Keywords:** hot spots, protein-DNA, EMD, CatBoost

## Abstract

Protein-DNA complex interactivity plays a crucial role in biological activities such as gene expression, modification, replication and transcription. Understanding the physiological significance of protein-DNA binding interfacial hot spots, as well as the development of computational biology, depends on the precise identification of these regions. In this paper, a hot spot prediction method called EC-PDH is proposed. First, we extracted features of these hot spots’ solid solvent-accessible surface area (ASA) and secondary structure, and then the mean, variance, energy and autocorrelation function values of the first three intrinsic modal components (IMFs) of these conventional features were extracted as new features via the empirical modal decomposition algorithm (EMD). A total of 218 dimensional features were obtained. For feature selection, we used the maximum correlation minimum redundancy sequence forward selection method (mRMR-SFS) to obtain an optimal 11-dimensional-feature subset. To address the issue of data imbalance, we used the SMOTE-Tomek algorithm to balance positive and negative samples and finally used cat gradient boosting (CatBoost) to construct our hot spot prediction model for protein-DNA binding interfaces. Our method performs well on the test set, with AUC, MCC and F1 score values of 0.847, 0.543 and 0.772, respectively. After a comparative evaluation, EC-PDH outperforms the existing state-of-the-art methods in identifying hot spots.

## 1. Introduction

The interactions between proteins and DNA are essential for numerous biological processes, such as self-replication and the transcription of nucleic acids, gene regulation [1,2] and transcription. At the interfaces of these interactions, some residues are considered hot spots, which contribute more binding free energy [3]. The identification of hot spots is of great significance for studying the mechanism of protein-DNA interactions and related biological explanations. Alanine scanning mutagenesis is considered a traditional experiment-based method used to identify hot spot residues and has long been used to identify these hot spots and explore protein-DNA recognition mechanisms [4]. However, this experiment-based method is time-consuming, labor-intensive and expensive, which makes computation-based methods the hot new methods for forecasting [5].

There are currently two main computation-based approaches being used to identify protein-DNA complexes: one utilizes molecular mechanics, and the other utilizes machine learning or deep learning models. SAMPDI [6] and PremPDI [7] are computational approaches based on molecular mechanics, and they predict changes in the free energy of protein-DNA binding. SAMPDI-3D [8], a gradient-boosting decision tree machine learning method was also used to predict the energy changes associated with both binding proteins and DNA base variants. Machine learning methods, including PrPDH [9] and WTL-PDH [10], focus on identifying hot spot residues at the protein-DNA binding interface or predicting hot spots through extensive attribute extraction. InpPDH [11] implements an SVM-based prediction model, employing both traditional and novel neighboring interface attribute features. Moreover, sxPDH [12] combines supervised isometric feature mapping with extreme gradient boosting based on PrPDH feature extraction to predict hot spot regions. SPDH [13] is a computational method based on amino acid sequence features, which extracts features from various contexts and then uses sequential forward selection to filter out the best subset of features to construct a predictive model. Similarly, PreHots [14] assembles a diverse dataset, reduces dimensionality using SFS, and an integrated classifier is then used to identify hot spots. Both energy-related features and structural features are introduced into the integrated models of PEMPNI [15], which predict the binding free energy changes of individual mutations. While effective, machine learning approaches present some challenges such as small and uneven datasets and underutilize valid information from traditional features. Therefore, future works should address these issues to improve their predictive performance.

In this study, we manually collected and screened 339 mutations from four protein thermodynamics databases. To solve the problem of a limited number of hot spots, we chose the SMOTE-Tomek [16] algorithm to balance the positive and negative samples. Then, the empirical mode decomposition algorithm (EMD) [17] was applied to process the extracted basic features, and a set of inherent modal components (IMFs) was obtained, from which the mean, variance, energy and autocorrelation function values were extracted. Finally, we obtained the best subset of features in 11 dimensions using a two-step feature selection algorithm (mRMR-SFS) [18] and constructed a hot spot prediction model for protein-DNA binding interfaces using CatBoost [19].

**Figure 1:**
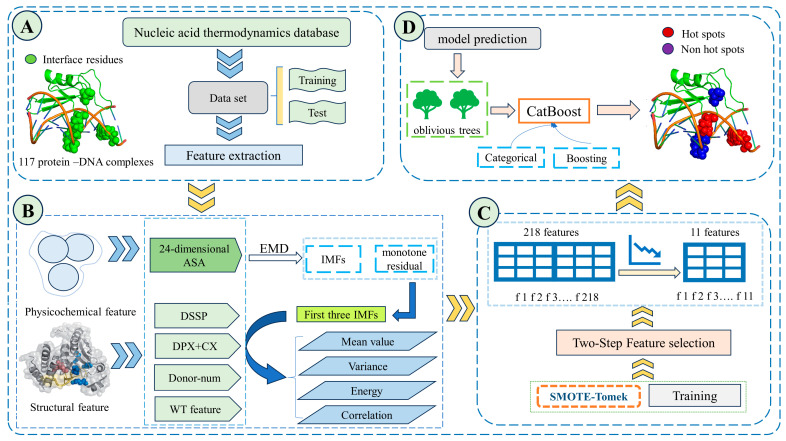
EC-PDH framework diagram. First, 117 complexes were manually collected and screened from four protein thermodynamic databases, and the characteristics of their ASA, uASA, dASA and secondary structure were extracted. After partitioning the dataset, the SMOTE-Tomek algorithm was selected to balance the positive and negative samples. Then, the empirical mode decomposition algorithm (EMD) was applied to process the extracted basic features, and the mean, variance, energy and autocorrelation function values were extracted from the first three intrinsic modal components (IMFs). Finally, a two-step feature selection method (mRMR-SFS) was used to obtain the optimal 11-dimensional subset of features, and CatBoost was used to construct a model to evaluate the test set. Part (**A**) is the data acquisition, processing and dataset partitioning. Part (**B**) is the extraction of physicochemical and structural features as well as the features of the IMF components obtained after processing by the empirical modal decomposition algorithm. Part (**C**) is the two-step feature selection algorithm and the SMOTE-Tomke feature balancing algorithm used in the training process. Part (**D**) is the prediction part of the trained CatBoost model, where red blobs indicate the correct hotspot residues of the model and the purple ones indicate the correctly predicted non-hotspot residues.

Our method performs well on the test set as its AUC, MCC and F1 scores are 0.847, 0.543 and 0.772, depending on the test set, and it demonstrates a superior performance compared to existing state-of-the-art methods in the prediction of hot spots at protein-DNA binding interfaces. The framework of the EC-PDH method is shown in Figure 1.

## 2. Materials and Methods

### 2.1. Datasets

Compared to our previous work on sxPDH [12], we have added two new data sources, one from Nabe [20] and the other from ProNAB [21]. We found 1627 mutated genes in 293 complexes. CD-HIT is a program developed in 2012 that can be used to cluster protein sequences, reduce sequence redundancy and improve the performance of sequence analysis, based on a novel parallelization strategy and several other techniques that allow the efficient clustering of such datasets [22]. To address the effect of redundant data, we first removed proteins with a sequence similarity greater than 40% using the CD-HIT tool, and then filtered out the interfacial residues with a soluble surface area greater than 1 Å using the NACCESS V2.1.1 [23] tool, which is a stand-alone program that calculates the soluble surface area of atoms and residues and is commonly used on amino acid and nucleic acid complexes.

According to the criteria of study [9], interface residues with ∆∆G≥1.0 kcal/mol are regarded as hot spots and the rest are regarded as non-hot spots. Thus, we extracted 131 hot spots and 208 non-hot spots from the 117 protein-DNA complexes as our dataset. Considering the small size of the dataset, which may lead to certain models exhibiting unexpectedly high performance, we performed 20 random divisions of the data to improve the reliability of the results, one of which is shown in Table 1. A total of 92 complexes were randomly selected to form the training set, which includes 102 hot spots and 179 non-hot spots. The other 25 complexes were used as the test set, which included 29 hot spots and 29 non-hot spots.

### 2.2. Feature Extraction

The identification of effective features of protein-DNA complexes can improve the accuracy of interface hot spots’ identification and can provide useful information to facilitate the interpretation of the mechanistic principles of protein-DNA interactions. In our previous work on WTL-PDH, we extracted 175 conventional dimensional features, including 43 dimensional physicochemical property features, secondary structure features and 132 dimensional discrete wavelet features. In this paper, we regarded the 43 dimensional physicochemical property features and secondary structure features as multiple non-smooth nonlinear signal sequences and extracted 43 new dimensional features using the empirical mode decomposition algorithm (EMD), which quantified 218 dimensional features.

#### 2.2.1. Solvent-Accessible Surface Area Characteristics

Solvent-accessible surface area features (ASAs) have been repeatedly shown to make a significant contribution to the identification of hot spots at the protein–nucleic acid binding interface [9,10,24,25,26]. We calculated absolute ASA values as well as relative ASA (RSA) values for all residues based on four of their atomic properties, namely, all-atom chains, nonpolar lateral chains, polar lateral chains, and full lateral chains, using NACCESS [23], and 8 dimensional ASA characteristics were obtained. ASA and RSA were subsequently calculated for these four properties in the residues’ singlet and complex states. The relative change in ASA or RSA from one state to another is regarded as a feature. A total of 24 ASA feature dimensions were visualized and quantified.

#### 2.2.2. Secondary Structure Features

To calculate the structural features of the proteins in the dataset, we used the protein secondary structure definition (DSSP) to characterize the secondary structure of the proteins [27], which includes their carbonyl angle, torsion angle, bond angle, and water molecule number [27]. In total, 6 dimensional features were summarized.

#### 2.2.3. Depth Index and Protrusion Index

The geometric complementarity of the binding interface is important for protein-DNA interactions [28]. Pintar et al. (2002) demonstrated that the depth index (DPX) and convexity index (CX) enhance the predictive performance of thermal residue models by using the depth index (DPX) and convexity index (CX) to characterize the embedding and protruding conditions of atoms surrounded by other non-hydrogen atoms, respectively [29]. We calculated two atomic attribute values and residues’ shrinkage and non-shrinkage states were analyzed using PSAIA [30], including the average of all their atoms and the standard deviation of their side-chain atoms, for a total of 8 dimensional features. After that, we also calculated the changes in DPX to CX relative to the two states, yielding a total of 4 dimensional features. A total of 12 measures of characterization were generated.

#### 2.2.4. Number of Hydrogen Bonds

Hydrogen bonding affects hot spot recognition at protein-DNA binding interfaces [9,14]. Here, we used the HBPLUS [31] tool to count the number of bonds in all the protein-DNA mixtures in the dataset to serve as the new feature. This section generated a total of 1 dimensional feature.

#### 2.2.5. Wavelet Transform Features

We processed four sets of features, the ASA, uASA, dASA and substructure, as a digital signal through the discrete wavelet transform (DTW) in MATLAB and wavelet packet transform (WPT) via the wavelet function db1. Ea (3 dimensions), Ea standard deviation (1 dimension), and Ea mean (1 dimension) were extracted for the end node of the wavelet packet tree at the third level, as were the Ed (1 dimension) and the above wavelet entropy characterization (5 dimensions), absolute energy summaries (1 dimension), absolute energy values (8 dimensions), wavelet entropy features (5 dimensions) and relative energy values (8 dimensions). In total, 4×33=132 dimensional features were quantified [10].

#### 2.2.6. EMD Feature

In our study, our identified method can decompose a complex signal into several intrinsic modal components (IMFs), characterizing the local properties of the signal. This method is adaptive to nonlinear and nonstationary signals, boasting the advantage of a high signal-to-noise ratio and excellent time–frequency focusing. This method has been utilized in the analysis of DNA sequence problems [32,33,34,35].

Here, we detail the steps involved in EMD feature extraction. Initially, we generate all the extreme points based on the original signals, subsequently creating the upper and lower envelopes. The mean value of the upper and lower envelopes is subsequently identified on a point-by-point basis, leading to the extraction of the mean value from the original signals and resulting in an intermediate signal. This signal will need to satisfy two conditions relating to the IMF, namely that, throughout the data range, the quantity of extreme value points and crossing zeros should be equal or differ by no more than one. Furthermore, at any given moment, the mean value stemming from the upper envelope (formed by high local value points) and the lower envelope (formed by low local value points) should be zero. Once the intermediate signal is designated as an IMF, it is extracted from the original signals, yielding a residual. This residual signal becomes the basis for repeating the process for the creation of additional IMFs.

Next, we created four IMF functions for ASA, uASA, dASA and secondary structure features, respectively, and we considered them digital signals and processed them to provide a spectrum of measurements, including the mean, variance, energy, and autocorrelation function value, yielding, in total, 43 dimensional features. The specific formulae are as follows:

(1) $$X^{-}=\frac{1}{N}\sum_{i=0}^{N-1}x_{i}$$

(2) $$S^{2}=\frac{1}{N-1}\sum_{i=0}^{N-1}{\left(x_{i}-meanValue\right)}^{2}$$

(3) $$E=\sum_{i=0}^{N-1}{\left|x_{i}\right|}^{2}$$

(4) $$Ac=\sum_{i=0}^{N-1}{x_{i}}^{*}\cdot x_{i+m}$$

where N is the length of each IMF digital signaling component, $x_{i}$ denotes the value taken at the ith moment in each digital signal, ${x_{i}}^{*}$ denotes the value taken at the ith moment in each digital signal, $x_{i+m}$ denotes the conjugate fetch value of the signal separated by m units from the $x_{i}$ signal fetch value. In total, 43 dimensional features are quantized. $X^{-}{,S}^{2},E\;and\;Ac\;$ represent the mean, variance, energy and autocorrelation function values, respectively.

### 2.3. Data Balancing

In the dataset, the number of positive samples (hot spots) is relatively small, which may affect the generalization ability of the model and its prediction accuracy during the training process. In addition, the conventional synthetic minority oversampling technique (SMOTE) is extremely sensitive to noise and outliers. Therefore, we chose to use an improved integrated sampling strategy, the SMOTE-Tomek algorithm, to generate a balanced dataset.

The method used in this paper extends the dataset by first applying the SMOTE algorithm to create new negative samples during training. Subsequently, the Tomek Link algorithm is run on the expanded dataset to find and remove instances belonging to the largest category. This approach creates clearer categorization boundaries and thus improves the prediction accuracy of the model. The specific steps to realize this are as follows: first, the new samples are calculated by the formula below.

(5) $$X_{new}=X+\left|X-X_{n}\right|\times rand\left(0,1\right)$$

Oversampling generates a small number of samples, where $X_{new}$ is the newly generated sample and  X is each of the non-hot spot samples. $X_{n}$ denotes a randomly selected sample from n neighboring samples and $rand\left(0,1\right)$ indicates a random number greater than 0 and less than 1. Then, Tomek links are applied to reduce the number of majority class samples at the boundary, which makes the number of majority class samples close to the number of minority class samples. In some cases, it may be possible to directly remove samples close to the boundary because, after oversampling, the ratio of hot spot samples to non-hot spots samples reaches 1:1. In this way, the impact of sample imbalance can be significantly reduced. In this process, we set the random seed parameter random_rate to 74 for comparison to previous experiments.

### 2.4. Feature Selection

In our previous data extraction work, we extracted a total of 218 dimensional features, including physical and chemical property features, secondary structure features, wavelet transform features and EMD features. When there are too many feature dimensions, model overfitting may occur; the accuracy and generalizability of the model can be significantly improved, while the risk of overfitting can be reduced by eliminating irrelevant or redundant features and retaining the most relevant features in the model. Moreover, feature selection also reduces computational complexity and storage space, speeds up training and reduces the time consumed by computational resources. Previous studies have shown that a hybrid two-step feature selection strategy can be used to effectively filter out a subset of relevant features. In this work, we first use the maximum relevance minimum redundancy method (mRMR) [36] to rank the importance of 218 features. In the second step, the mRMR-ranked feature vectors are processed using the sequential forward feature selection (SFS) [37] method to obtain the optimal 11-dimensional-feature subset, as shown in Table 2.

We also compare this outcome with other popular feature selection approaches. These include Random Forest (RF) [38], mRMR, SVM-based Recursive Feature Elimination (SVM-RFE) [39], SFS and sequential forward-selection-based RF (RF-SFS) [40]. We then relied on the 11-dimensional subset of the best features extracted by the two-step feature selection algorithm to identify hot spots at the protein-DNA complex interface.

### 2.5. Model Construction

CatBoost (categorical boosting) is a Yandex open source machine learning algorithm that can handle multiple data types [19]. XGBoost [41] and LightGBM [42,43] are the three mainstream GBDT [44] tools in the GBDT algorithm framework with improved implementation. CatBoost is based on a symmetric decision tree (oblivious trees) and is the basis of the learner implementation of the GBDT framework, with fewer parameters and support for categorical variables. CatBoost is composed of the terms categorical and boosting, which mainly address its main point: efficiently and rationally processing categorical features. In addition, CatBoost addresses the problems of gradient bias and prediction shift to reduce the occurrence of overfitting and thus improve the accuracy and generalizability of algorithms [42,45]. In this study, we used CatBoost to train our model and obtained the optimal parameters for the model, which are as follows: maximum number of iterations = 150, learning rate = 0.03, tree depth = 6 and random_seed = 42.

### 2.6. Performance Evaluation

We performed simultaneous feature selection on the training set using ten-fold cross-validation to attain the optimal feature subset and adjust the parameters of the CatBoost model to maximize hot spot recognition accuracy. Our method uses 50 10-fold cross-validation results on the training dataset, as shown in Table 3. In order to evaluate the performance of our constructed model, we used some common evaluation metrics for binary classification tasks, including Sensitivity (*SEN*), Specificity (*SPE*), Precision (*PRE*), F1 Score (*F*_1_) and Accuracy (*ACC*), as well as Matthew’s Correlation Coefficient (*MCC*). The definitions of these metrics are as follows:

(6) $$SEN=\frac{TP}{TP+FN}$$

(7) $$SPE=\frac{TN}{TN+FP}$$

(8) $$PRE=\frac{TP}{TP+FP}$$

(9) $$F_{1}=\frac{2\times SEN\times PRE}{SEN+PRE}$$

(10) $$ACC=\frac{TP+TN}{TP+TN+FP+FN}$$

(11) $$MCC=\frac{TP\times TN-FP\times FN}{\sqrt{\left(TP+FP\right)\left(TP+FN\right)\left(TN+FP\right)\left(TN+FN\right)}}$$

Here, *TP*, *FP*, *TN* and *FN* denote the number of true positives (correct prediction of hot spot residues), false positives (non-hot spot residues incorrectly predicted to be hot spots), true negatives (correct prediction of non-hot spot residues) and false negatives (hot spot residues incorrectly predicted to be non-hot spots), respectively. For the sake of completeness, we also computed the area under the ROC curve (AUC) to assess the predictive performance of the model.

## 3. Results

### 3.1. Comparison of Various Data-Balancing Approaches

We compared the performance of the model on the training set under five conditions: using SMOTE-Tomek, SMOTE [46], the adaptive synthesis algorithm (ADASYN) [47], the random repeated oversampling balancing algorithm (simple replication operation) and without a data-balancing process. As shown in Table 4, the performance of the models trained with balanced data is substantially improved compared to the model trained with unprocessed data. The high number of negative samples in the untreated dataset leads to a biased prediction of negative samples by the model. The AUC of this model is only 0.721, with poor generalization ability. However, after the SMOTE-Tomek operation, the AUC of the model is improved to 0.859 and its F1 score is improved to 0.769. During our comprehensive evaluation, we used the SMOTE-Tomek method to equalize the number of positive and negative samples in our dataset in order to construct our model.

### 3.2. Comparison of Various Feature Selection Approaches

We compared six common feature selection approaches using CatBoost-based classification models, namely mRMR, the SFS, the RF, the SVM-RFE, the mRMR-SFS and the RF-SFS. As shown in Table 5, the PRE and SPE metrics are the best metrics in the model constructed based on the RF-SFS feature selection method, but all other metrics are clearly the highest in the model constructed based on the mRMR-SFS feature selection method, with its AUC even reaching the highest value of 0.859. The mRMR-SFS method first filters a set of features from the total set of features that are most relevant to the sample labels but most irrelevant to each other, after which it ranks them. The SFS method involves iteratively adding subsets of features to optimize model performance. Taken together, we used the mRMR-SFS feature selection method to construct our model.

### 3.3. Importance Ranking of Features and the Best Subset

**Figure 2:**
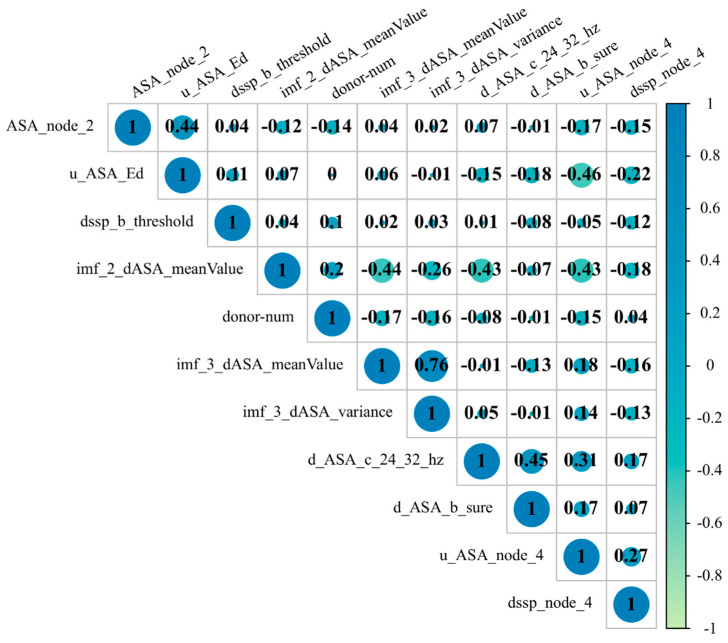
Correlation heatmap between the features of the best feature subset.

Using the mRMR-SFS feature selection method, we have identified the best subset of 11 dimensional features, as shown in Table 2. Among these features, three were extracted through EMD. In order to assess the contribution of different classes of features to the model’s predictive performance, we plotted a correlation heatmap between the feature subsets, as shown in Figure 2.

**Figure 3:**
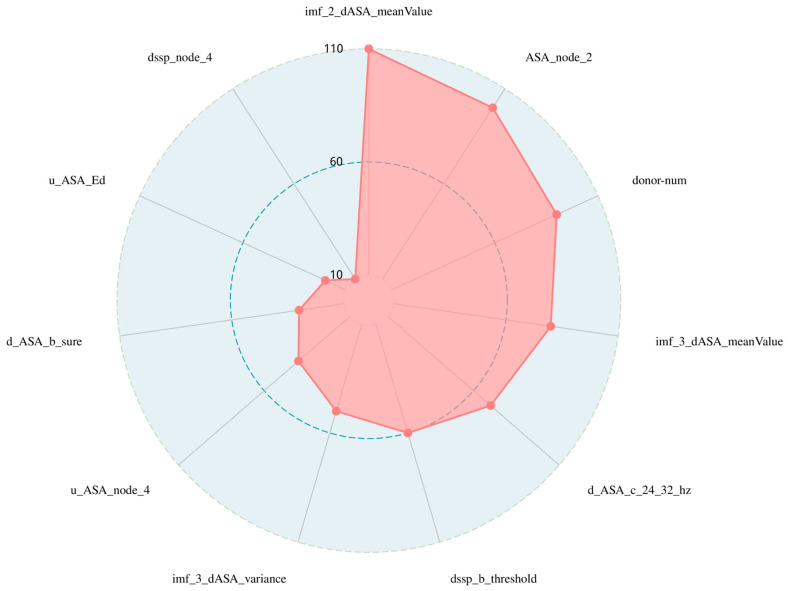
Radar chart of features’ importance in the best feature subset. This is a radar plot of the importance of the 11-dimensional subset of the best features filtered by the two-step feature selection. In order to make the results more intuitive, we quantify the importance values of the 11-dimensional best features in the graph, with higher red values indicating greater importance, and it can be seen that the importance of these features for identifying hotspots decreases in a clockwise direction.

Moreover, as shown in the radar chart in Figure 3 and in Table 6, the EMD features contribute most to the identification of hot spot residues in protein-DNA complexes, and the combination of EMD features and wavelet features results in the best performance of the model. We believe that this is because the features extracted from the IMF contain more information, which can supplement the information carried by the wavelet features. Therefore, more hot spot feature information can be represented with fewer feature dimensions, providing a new feature extraction idea for identifying hot spots. These results suggest that EMD is a supplement to other types of characterization and helps to forecast hot spots in protein-DNA complexes.

### 3.4. Comparison of Different Classification Models

**Figure 4:**
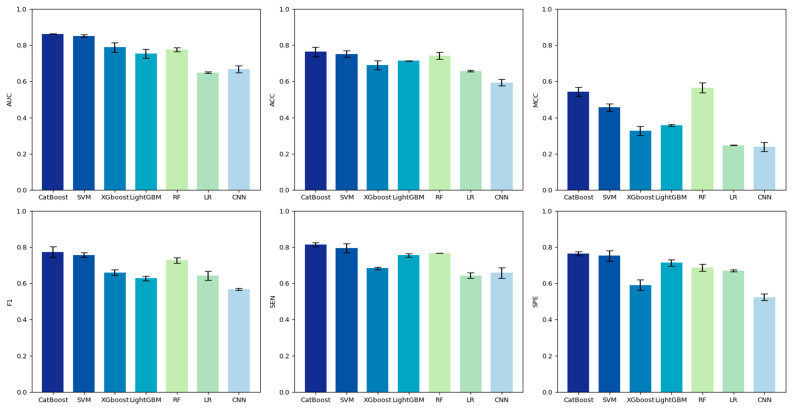
Comparison of the performance of models constructed with various classifiers used on the training set.

In order to obtain the best hot spot recognition model, we comprehensively compared the performance of classifiers such as CatBoost, LightGBM, XGBoost, logistic regression (LR), SVM (Using the sigmoid kernel function), RF and CNN on the training set. Figure 4 shows the performance of six machine learning classifiers and one deep learning classifier on the training set. A performance comparison with 10-fold cross-validation showed that CatBoost significantly outperformed the other six classifiers using the training set (*SEN* = 0.764, *SPE* = 0.756, *F*_1_ = 0.772, *MCC* = 0.543, *ACC* = 0.764, *AUC* = 0.863). While RF has a slightly better MCC, the CatBoost model is more suitable for constructing our model when considering the performance metrics comprehensively.

### 3.5. Performance Comparison of Different Methods on the Test Set

**Figure 5:**
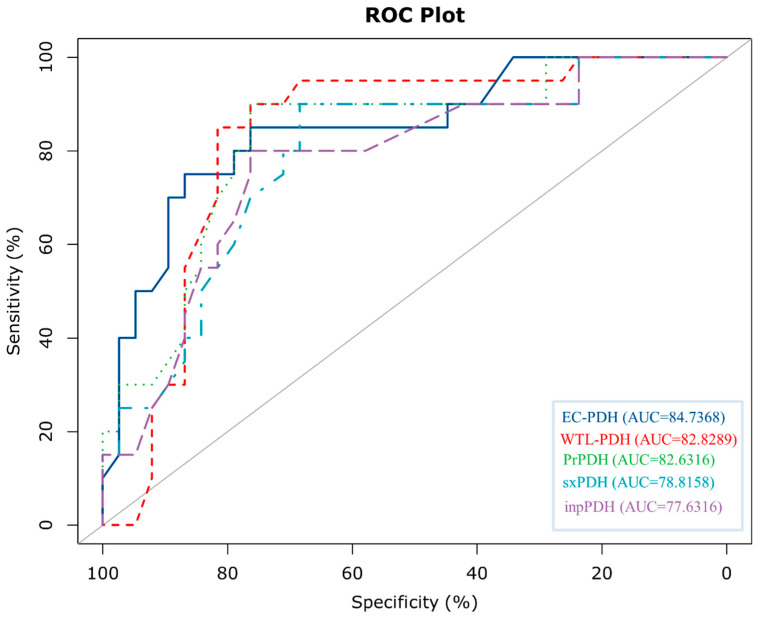
Comparison of ROC curves plotted by various methods for the test set.

**Figure 6:**
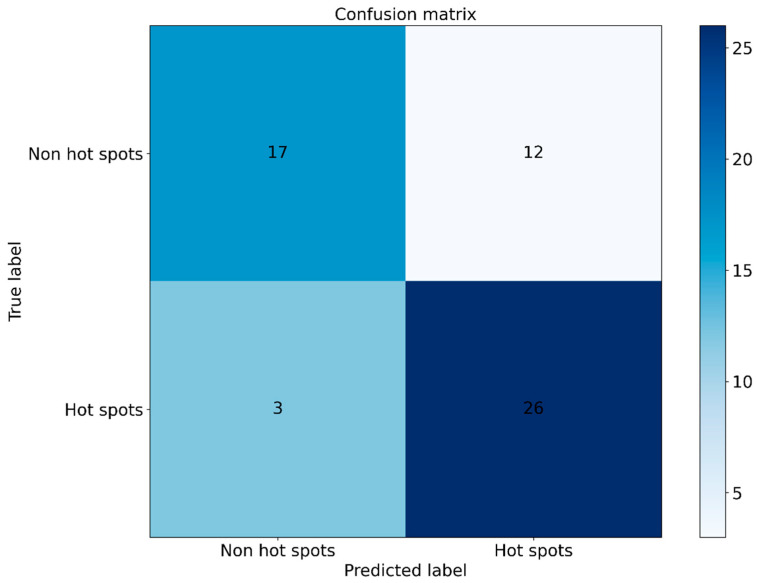
Confusion matrix plot for the test set.

To further evaluate the generalization ability of EC-PDH, we compared it with state-of-the-art methods, including WTL-PDH, PrPDH, sxPDH and inpPDH. Figure 5 shows the ROC curves of the above five methods on the test set. It can be seen that our method, with an *AUC* = 0.847, has the best prediction performance and generalization ability. Furthermore, we plotted the confusion matrix of EC-PDH for the test set, as shown in Figure 6. For the 58 samples in the test set, our method successfully predicted 43 hot spots and non-hot spots and misidentified 3 hot spots and 12 non-hot spots.

### 3.6. Case Study

**Figure 7:**
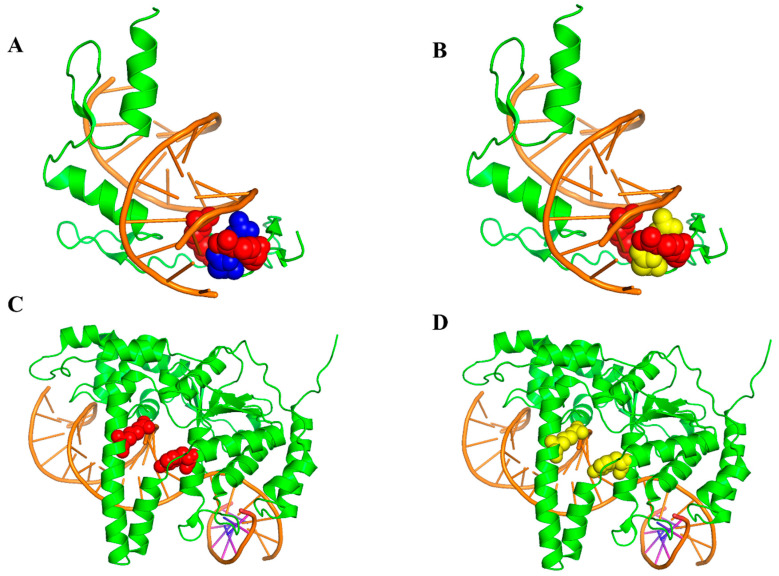
Identified hot and non-hot spots in 1AAY using the EC-PDH (**A**) and PrPDH (**B**) methods. The EC-PDH (**C**) method and PrPDH (**D**) method were used to identify hot and non-hot spots in 3Q8L. The color scheme chosen is as follows: orange for DNA sequences and green for protein sequences. Red indicates correctly identified hot spot residues and purple indicates correctly identified non-hot spot residues. Yellow indicates residues that were incorrectly predicted (hot and non-hot spots).

The Zif268 Protein-DNA complex (PDB ID: 1AAY, A chain) has been shown to be a system that can aid in the recognition of DNA by using zinc from TFIiA [48]. There are two hot spots (R118 and R124) and two non-hot spots (D120 and E121) in the complex. The predictive results, procured using EC-PDH and PrPDH, are delineated in Figure 7. As shown in Figure 7A,B, EC-PDH successfully predicted all hot and non-hot spots, while two non-hot spot residues (D120 and E121) were inaccurately predicted by PrPDH. Subsequently, we investigated the crystallographic structure of the human valvular endonuclease FEN1 (WT) in complex with the substrates 5′-flap DNA, SM3+ and K+ (PDB ID: 3Q8L, chain A), which include two hot spots in their protein chain (Y40 and R100) [49]. In Figure 7C,D, while EC-PDH accurately recognized the two hot spots, all judgments rendered by PrPDH were incorrect.

## 4. Conclusions

In this study, we developed a new method, named EC-PDH, for identifying hot spots at protein-DNA binding interfaces. In the initial phase of the study, we screened 1627 mutations from four protein thermodynamics databases and divided them into a training set and a test set. To address the problem of sparse hot spot samples in the training set, we used the SMOTE-Tomek algorithm to balance positive and negative samples, thus optimizing the structure of the dataset. In the feature extraction stage, we obtained the solvent-accessible surface area (ASA) and secondary structure characteristics and identified the intrinsic modal components (IMFs) of these basic features by means of an empirical modal decomposition algorithm (EMD). We selected the first three intrinsic modal components and extracted their four attributes, mean, variance, energy and autocorrelation function values, to obtain a total of 218 features. Next, we filtered the features in the training phase using the maximum correlation minimum redundancy sequence forward-selection method (mRMR-SFS) to obtain the best subset of 11 dimensional features. Finally, we utilized the CatBoost algorithm as a classifier to predict hot spots and non-hot spots at protein-DNA binding interfaces. Our method demonstrates an efficient prediction performance on the test set, which positively contributed to the advancement of research on protein-DNA interaction mechanisms. We believe that the technological innovation brought about by EC-PDH will have a positive impact on future biological research.

## Figures and Tables

**Table 1:** One of the randomly divided datasets.

Dataset	Number of Variants	Amount of PDBs	Number of Hot Spots	Number of Non-Hot Spots	Ratio
Training	271	92	102	179	0.569
Test	68	25	29	29	0.500

The ratio represents the ratio of the number of positive to negative samples in the training and test sets.

**Table 2:** Ranking of the importance of the 11 best features.

Number	Feature	Feature Description
1	imf_2_dASA_meanValue	The mean of the second IMF digital signaling component after the EMD of dASA
2	ASA_node_2	The baud sign of the second node of the third layer of ASA after WPT processing
3	donor-num	Number of hydrogen bonds
4	imf_3_dASA_meanValue	The mean of the third IMF digital signaling component after the EMD of dASA
5	d_ASA_c_24_32_hz	Absolute energy of the eighth node after WPT processing by uASA
6	dssp_b_threshold	Threshold of secondary structural features after WPT
7	imf_3_dASA_variance	The variance of the third IMF digital signaling component after the EMD of dASA
8	u_ASA_node_4	uASA: the baud sign of the fourth node of the third layer after WPT processing
9	d_ASA_b_sure	Shannon entropy of dASA features after WPT
10	u_ASA_Ed	The Ed of the third layer wavelet approximation coefficient is obtained using the uASA wavelet transform
11	dssp_node_4	DSSP: the baud sign of the fourth node of the third layer after WPT processing

Three of these are EMD features.

**Table 3:** EC-PDH 50 10-fold validation results on the training dataset.

Method	*SEN*	*SPE*	*PRE*	*F*_1_	*MCC*	*ACC*	*AUC*
EC-PDH	0.808	0.761	0.752	0.769	0.540	0.762	0.859

**Table 4:** Comparison of the performance of various data-balancing approaches on the training set.

Approach	*SEN*	*SPE*	*PRE*	*F1*	*MCC*	*ACC*	*AUC*
SMOTE-Tomek	**0.808**	0.761	**0.752**	**0.769**	**0.540**	**0.762**	**0.859**
SMOTE	0.782	**0.779**	0.748	0.762	0.531	0.758	0.848
ADASYN	0.721	0.572	0.653	0.612	0.351	0.651	0.735
Random Repetitive Oversampling	0.688	0.611	0.658	0.642	0.465	0.712	0.762
Unprocessed	0.189	0.645	0.364	0.266	0.324	0.642	0.721

Highest value is shown in bold in each column.

**Table 5:** Comparison of the performance of various feature selection approaches on the training set.

Approach	*SEN*	*SPE*	*PRE*	*F1*	*MCC*	*ACC*	*AUC*
mRMR-SFS (11)	**0.808**	0.761	0.752	**0.769**	**0.540**	**0.762**	**0.859**
mRMR (12)	0.705	0.724	0.737	0.708	0.465	0.713	0.789
SFS (15)	0.678	0.701	0.721	0.692	0.429	0.695	0.776
RF-SFS (16)	0.756	**0.762**	**0.756**	0.745	0.536	0.728	0.841
RF (21)	0.751	0.714	0.741	0.736	0.512	0.745	0.832
SVM-RFE (22)	0.681	0.649	0.624	0.526	0.215	0.621	0.694

The highest value is shown in bold in each column. The figures in parentheses represent the dimensions of the best subset of features selected.

**Table 6:** Effect of the combination of EMD and wavelet transform features on the model’s performance on the training set.

Feature Combination	*SEN*	*SPE*	*PRE*	*F*_1_	*MCC*	*ACC*	*AUC*
EMD and wavelet transform features	**0.813**	0.764	**0.756**	**0.772**	**0.543**	**0.764**	**0.863**
Wavelet transform features	0.784	**0.779**	0.746	0.765	0.533	0.754	0.847
EMD features	0.801	0.765	0.744	0.769	0.539	0.766	0.855

The highest value is shown in bold in each column. The feature combination indicates whether the feature is present in the model before feature selection.

## Data Availability

The original contributions presented in the study are included in the article, further inquiries can be directed to the corresponding author.
